# Giant Primary Epithelial Splenic Cyst in an Adolescent Girl

**DOI:** 10.21699/ajcr.v7i5.485

**Published:** 2016-11-01

**Authors:** Pradeep Kajal, Kamal Nain Rattan, Namita Bhutani, Kapil Bhalla

**Affiliations:** Department of Pediatric Surgery, PGIMS Rohtak, Haryana

**Dear Sir,**

A 13-year old girl presented with abdominal pain and upper abdominal fullness for 4 months. On examination, there was a large, smooth, and firm lump palpable involving the left hypochondrium, epigastrium, left lumbar region, and reaching almost up to the umbilicus. It was moving with respiration and the upper limit was not reachable. Rest of the examination was normal. All routine hematological and biochemical investigations were within normal range. Hematological tests for human immunodeficiency virus (HIV), hepatitis C virus (HCV) and hepatitis B virus (HBV) viz. ELISA for HIV, anti-HCV and HBsAg were negative. X-ray chest revealed slight elevation of left diaphragm. Abdominal ultrasonography revealed a giant unilocular cystic lesion in the spleen. Casoni’s skin test and complement fixation test for Echinococcus granulosus were negative. Stool examination done over 3 consecutive days was found to be normal. Computed tomography confirmed the splenic large cyst with dimensions of 22x17x15cm with thin, compressed rim of normal parenchyma posterolaterally (Fig.1). At laparotomy, a huge splenic cyst of more than 20 cm of maximum diameter was found with almost total displacement and compression of remaining splenic parenchyma. However due to cyst size and location, preservation of spleen was considered impossible and total splenectomy was carried out after decompression of the cyst by aspiration of about 2000 ml of turbid serous fluid (Fig.2). The postoperative period was uneventful and patient was discharged in fair health on 5th post-operative day. The histopathological examination of excised specimen revealed the cyst lined by tubulocolumnar, pseudostratified (mesothelial line) epithelium. Focally, the lining epithelium was flattened and the cyst wall was composed of fibrocollagenous tissue. The cyst was filled with eosinophillic material and foamy and hemosiderin-laden macrophages. Aspirated cystic fluid showed no evidence of malignancy. Patient had received Hemophilus influenza, Pneumococcal (unconjugated 23 valent) and meningococcal vaccine 2 weeks before surgery and antibiotic prophylaxis was given after surgery for a period of 6 months. The patient is on regular follow up for last one year and doing very well.

**Figure:**
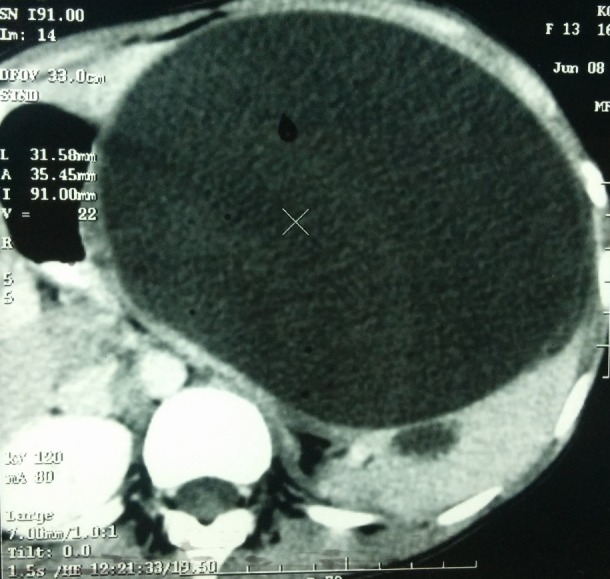
Figure 1:CT scan demonstrating a very large, homogeneous splenic cyst compressing the normal splenic parenchyma and adjacent viscera and even crossing the midline.

**Figure:**
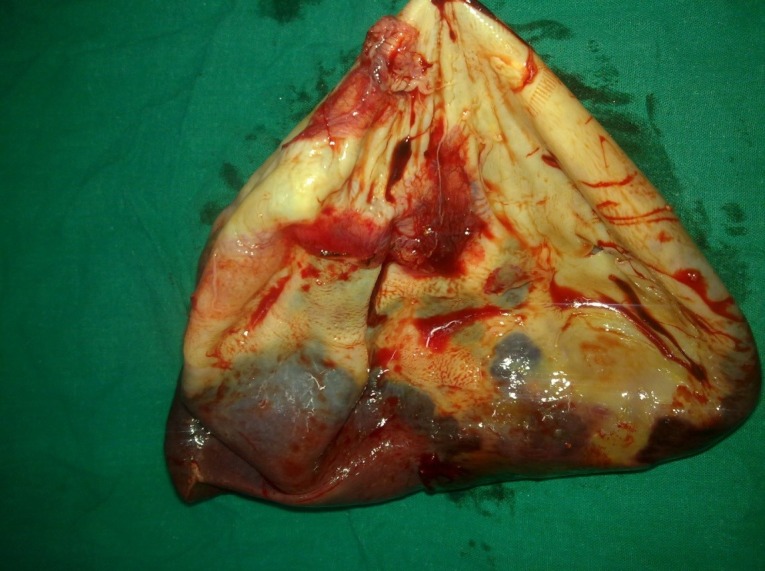
Figure 2:Resected specimen of giant splenic cyst after decompression.

Splenic cysts are as type 1 cysts, which are true cysts having lining epithelium, and type 2 cysts, which are false cysts without lining epithelium.[1] Depending on the causative agent splenic cysts can be divided into parasitic and non-parasitic cysts. Non-parasitic splenic cysts are further classified as congenital, neoplastic, traumatic, and degenerative.[2] Primary splenic cysts constitute 10% of all nonparasitic cysts of the spleen. Primary epithelial splenic cyst is a rare condition with an incidence rate of 0.07%.[3].

The pathogenesis of primary splenic cysts is not clear. Splenic cysts are predominantly seen in the second and third decades; however, they can be seen in paediatric age group. Usually small cysts are asymptomatic. A painless mass in the left hypochondrium is the main presentation in 30%-40% cases. There may be localized pain or referred pain due to mass effect in case of bigger cysts. Occasionally the patients may present with thrombocytopenia. Occasionally they present with complications like infection, rupture and haemorrhage.[4] Primary epithelial cysts are usually solitary, but can be multiple.

Previously, splenectomy was the treatment of choice for splenic cysts.[5] Other options are percutaneous aspiration or percutaneous drainage,[5] partial splenectomy with a stapler or harmonic scalpel, total cystectomy, marsupialization or cyst de-roofing,[6] laparoscopic puncture and creation of a cyst-peritoneal window.[7] In case of total splenectomy, vaccination, and long-term antibiotic prophylaxis is given to protect against post-splenectomy overwhelming infections.

## Footnotes

**Source of Support:** Nil

**Conflict of Interest:** None declared

